# Gesundheit von Menschen mit ausgewählten Staatsangehörigkeiten in Deutschland: Prävalenzen nichtübertragbarer Erkrankungen und damit assoziierte soziale sowie migrationsbezogene Faktoren

**DOI:** 10.1007/s00103-023-03767-4

**Published:** 2023-09-20

**Authors:** Susanne Bartig, Marleen Bug, Carmen Koschollek, Katja Kajikhina, Miriam Blume, Manuel Siegert, Christin Heidemann, Lena Walther, Hannelore Neuhauser, Claudia Hövener

**Affiliations:** 1 Abteilung für Epidemiologie und Gesundheitsmonitoring, Robert Koch-Institut, Berlin, Deutschland; 2 Abteilung für Infektionsepidemiologie, Robert Koch-Insitut, Berlin, Deutschland; 3 Forschungszentrum des Bundesamts für Migration und Flüchtlinge, Nürnberg, Deutschland

**Keywords:** Migration, Soziale Determinanten, Gesundheitliche Ungleichheit, Chronische Erkrankungen, Depression, Migration, Social determinants, Health inequality, Chronic diseases, Depression

## Abstract

**Hintergrund:**

Gesundheitliche Chancen und Risiken werden durch eine Vielzahl von Faktoren beeinflusst. Der Beitrag zielt darauf ab, die Gesundheit von Menschen mit ausgewählten Staatsangehörigkeiten anhand nichtübertragbarer Erkrankungen (chronische Krankheit oder lang andauerndes gesundheitliches Problem allgemein, koronare Herzkrankheit, Diabetes mellitus, Depression) zu beschreiben und assoziierte Faktoren zu identifizieren.

**Methoden:**

Die Analysen basieren auf Daten der multimodalen, mehrsprachigen Befragungsstudie „Gesundheit in Deutschland aktuell: Fokus“ (GEDA Fokus), die unter 18- bis 79-Jährigen mit italienischer, kroatischer, polnischer, syrischer oder türkischer Staatsangehörigkeit deutschlandweit durchgeführt wurde (11/2021–05/2022). Um Zusammenhänge zwischen den nichtübertragbaren Erkrankungen und sozialen sowie migrationsbezogenen Merkmalen zu untersuchen, wurden Prevalence Ratios und 95 %-Konfidenzintervalle mittels Poisson-Regressionen berechnet.

**Ergebnisse:**

Insbesondere ein geringes Zugehörigkeitsgefühl zur Gesellschaft in Deutschland sowie selbstberichtete Diskriminierungserfahrungen im Alltag sind mit höheren Prävalenzen einer chronischen Erkrankung oder eines lang andauernden gesundheitlichen Problems und – gemäß selbstberichteten ärztlichen Diagnosen – mit einer Depression sowie zum Teil mit einer koronaren Herzerkrankung und einem Diabetes assoziiert.

**Diskussion:**

Die Ergebnisse verweisen auf gesundheitliche Ungleichheiten unter Menschen mit ausgewählten Staatsangehörigkeiten, die – vor dem Hintergrund der Bedeutung des subjektiven Zugehörigkeitsgefühls zur Gesellschaft in Deutschland und der selbstberichteten Diskriminierungserfahrungen für die betrachteten nichtübertragbaren Erkrankungen – möglicherweise auf eingeschränkte Teilhabechancen und gesellschaftliche Ausschlussmechanismen hindeuten.

## Abstract (en)

**Background:**

Health chances and risks of people with a history of migration vary according to a wide range of factors. This paper aims to describe the health of people with selected citizenships on the basis of four non-communicable diseases (chronic disease or long-term health problem in general, coronary heart disease, diabetes mellitus, depression) and to identify associated social and migration-related factors.

**Methods:**

Analyses are based on data from the multilingual and multimodal interview survey “German Health Update: Fokus” (GEDA Fokus), which was conducted among 18- to 79-year-olds with Croatian, Italian, Polish, Syrian, or Turkish citizenship living in Germany (November 2021 to May 2022). Poisson regressions were used to calculate prevalence ratios and 95% confidence intervals to examine the association between the individual indicators and social as well as migration-related characteristics.

**Results:**

In particular, a low sense of belonging to the society in Germany and self-reported experiences of discrimination in everyday life are associated with higher prevalence of a chronic disease or long-term health problem and – according to self-reported medical diagnoses – with depression and partly with coronary heart disease and diabetes.

**Discussion:**

Given the importance of subjective sense of belonging to the society in Germany and self-reported experience of discrimination for the health outcomes studied, the results point to health inequalities among people with selected citizenships that may indicate mechanisms of social exclusion.

## Einleitung

Eine wesentliche Voraussetzung für die Teilhabe von Menschen in verschiedenen Lebensbereichen stellt ihre Gesundheit dar, die in enger Wechselwirkung mit den Lebensphasen und -lagen steht (z. B. Alter, sozioökonomischer Status, Wohn- sowie Arbeitsbedingungen). Die gesundheitliche Situation von Menschen mit (eigener oder familiärer) Migrationsgeschichte wird durch verschiedene Faktoren vor, während und nach der Migration beeinflusst [1–3]. Demzufolge sind Gesundheitsressourcen sowie Krankheitsrisiken nicht allein auf die Migration an sich zurückzuführen, sondern es bedarf der Berücksichtigung der Lebensumstände und der damit einhergehenden Zugangschancen zu gesellschaftlichen Ressourcen, wie dem Gesundheitssystem, aber auch dem Arbeitsmarkt oder dem Bildungssystem. So variieren die gesundheitlichen Chancen und Risiken innerhalb der Bevölkerung mit Migrationsgeschichte unter anderem nach den Motiven sowie Umständen des (eigenen oder familiären) Migrationsprozesses, der Aufenthaltsdauer, dem Aufenthaltsstatus, den Ausgrenzungs- und Diskriminierungserfahrungen, den Kenntnissen der deutschen Sprache, dem sozioökonomischen Status sowie den Lebens- und Arbeitsbedingungen [2, 4].

Vor diesem Hintergrund ist es unerlässlich, diese große Heterogenität in Analysen und bei der Berichterstattung zu Migration und Gesundheit zu berücksichtigen. Allerdings gibt es in Deutschland nur wenige Datenquellen, in denen Menschen mit Migrationsgeschichte ausreichend repräsentiert sind, um differenzierte Aussagen zur gesundheitlichen Lage nach soziodemografischen, sozioökonomischen sowie migrationsbezogenen Merkmalen zu ermöglichen. Während in den letzten Jahren das wissenschaftliche Interesse insbesondere hinsichtlich der psychischen Gesundheit [5–9] und der Inanspruchnahme von Leistungen des Gesundheitssystems [10–13] zunahm, mangelt es an empirischen Erkenntnissen zur körperlichen Gesundheit von in Deutschland lebenden Menschen mit Migrationsgeschichte.

Nichtübertragbare Erkrankungen (*non-communicable* diseases, NCDs) sind die weltweit häufigsten Todesursachen: 74 % der weltweiten Todesfälle sind auf NCDs zurückzuführen [14], in Deutschland sogar 85 %. Mehr als drei Viertel (80 %) aller vorzeitigen Todesfälle infolge von NCDs sind – laut der Weltgesundheitsorganisation (WHO) – durch die „world’s biggest killers“ [15] Herz-Kreislauf-Erkrankungen, Krebs- und Atemwegserkrankungen sowie Diabetes mellitus bedingt [14]. Trotz der hohen Public-Health-Relevanz dieser „main types of NCDs“ mangelt es an aktuellen, repräsentativen Erkenntnissen für in Deutschland lebende Menschen mit Migrationsgeschichte. Bisherige Studien fokussieren insbesondere Inzidenz- und Mortalitätsraten von Krebserkrankungen, die größtenteils auf spezifische Regionen in Deutschland und bestimmte Herkunftsgruppen begrenzt sind [16–19].

Der vorliegende Beitrag zielt darauf ab, die gesundheitliche Situation von Menschen mit italienischer, kroatischer, polnischer, syrischer oder türkischer Staatsangehörigkeit anhand ausgewählter nichtübertragbarer Erkrankungen auf Basis der multimodalen, mehrsprachigen Befragungsstudie GEDA Fokus differenziert zu beschreiben und die mit den jeweiligen Erkrankungen assoziierten Faktoren zu identifizieren. Neben dem Vorliegen einer chronischen Krankheit oder eines lang andauernden gesundheitlichen Problems allgemein (für mindestens 6 Monate) werden drei ausgewählte selbstberichtete ärztliche Diagnosen zu chronischen Erkrankungen betrachtet: koronare Herzerkrankung, Diabetes mellitus und – zur Abbildung der psychischen Gesundheit – die Depression.

## Methoden

### Daten: Befragungsstudie GEDA Fokus

Die vorliegenden Analysen basieren auf Daten der mehrsprachigen Befragungsstudie „Gesundheit in Deutschland aktuell: Fokus“ (GEDA Fokus), die unter Menschen mit italienischer, kroatischer, polnischer, syrischer oder türkischer Staatsangehörigkeit im Alter von 18 bis 79 Jahren am Robert Koch-Institut (RKI) durchgeführt wurde. Ziel der Studie war es, umfassende Informationen zum Gesundheitszustand, Gesundheitsverhalten, den Lebensbedingungen und der Inanspruchnahme von Gesundheitsleistungen zu erheben und differenzierte Aussagen nach soziodemografischen, sozioökonomischen sowie migrationsbezogenen Merkmalen zu ermöglichen.

Mittels einer Einwohnermeldeamtsstichprobe wurden die Studienpersonen nach dem Merkmal Staatsangehörigkeit (1., 2. oder 3. Staatsangehörigkeit; entsprechend sind Personen mit doppelter Staatsangehörigkeit eingeschlossen) aus 99 Städten und Gemeinden in ganz Deutschland zufällig ausgewählt. Die Auswahl der fünf Staatsangehörigkeitsgruppen (Grundgesamtheit) erfolgte anhand von Modellrechnungen unter Verwendung der Ausländerstatistik des Statistischen Bundesamtes für die Jahre 2015 bis 2017 und unter Berücksichtigung der Gruppengröße sowie der Dynamik in Form von Zu- und Fortzügen [20]. Das Ziehungsmerkmal „Staatsangehörigkeit“ wurde gewählt, da dies das einzige migrationsbezogene Merkmal ist, das zuverlässig in den Einwohnermelderegistern erfasst ist.

Die Datenerhebung erfolgte sequentiell in einem Mixed-Mode-Design von November 2021 bis Mai 2022. Neben einer mehrsprachigen Onlinebefragung konnten die Studienpersonen über einen schriftlichen Papierfragebogen auf Deutsch oder in einer der fünf Studiensprachen (Arabisch, Italienisch, Kroatisch, Polnisch oder Türkisch) teilnehmen. Zudem bestand die Möglichkeit eines persönlichen^1^ oder telefonischen Interviews mit teilweise mehrsprachigen Interviewenden. Nähere Informationen zum Studiendesign von GEDA Fokus sind an anderer Stelle beschrieben [20].

Insgesamt nahmen 6038 Personen (2983 Frauen, 3055 Männer) an GEDA Fokus teil. Die Responserate betrug nach den Standards der American Association for Public Opinion Research (AAPOR) 18,4 % (Responserate 1; [21]).

### Indikatoren

Die Auswahl der nichtübertragbaren Erkrankungen erfolgte in Anlehnung an die „main types of NCDs“ der WHO [14] und das im Rahmen des Projektes IMIRA (Improving Health Monitoring in Migrant Populations; [22]) entwickelte (Kern‑)Indikatorenset zur Beschreibung der gesundheitlichen Lage von Menschen mit Migrationshintergrund [23]. Um der Heterogenität der Zielgruppe annähernd gerecht zu werden und mögliche Determinanten der jeweiligen Erkrankungen zu identifizieren, wurden verschiedene soziale sowie migrationsbezogene Merkmale berücksichtigt [24].

#### Nichtübertragbare Erkrankungen zur Beschreibung der gesundheitlichen Lage

Das Vorliegen einer chronischen Krankheit oder eines lang andauernden gesundheitlichen Problems wurde in GEDA Fokus mit der Frage erfasst: „Haben Sie eine chronische Krankheit oder ein lang andauerndes gesundheitliches Problem? Damit gemeint sind Krankheiten oder gesundheitliche Probleme, die mindestens 6 Monate andauern oder voraussichtlich andauern werden“ (Antwortkategorien: „Ja“, „Nein“). Neben der koronaren Herzerkrankung wurde das Vorliegen eines Diabetes mellitus über die selbstberichtete, jemals gestellte ärztliche Diagnose erhoben („Hat ein Arzt oder eine Ärztin bei Ihnen jemals [eine koronare Herzkrankheit^2^]/[eine Zuckerkrankheit oder einen Diabetes] festgestellt?“; Antwortkategorien: „Ja“, „Nein“, „weiß nicht“). Als Indikator für die psychische Gesundheit wurde die Lebenszeitprävalenz der selbstberichteten ärztlich diagnostizierten Depression ausgewählt („Wurde bei Ihnen jemals von einem Arzt oder einer Ärztin bzw. von einem Psychotherapeuten oder einer Psychotherapeutin eine Depression festgestellt?“; Antwortkategorien: „Ja“, „Nein“, „weiß nicht“). Alle Indikatoren wurden dichotomisiert; die Angabe „weiß nicht“ wurde als fehlender Wert aus den Analysen ausgeschlossen.

#### Soziale und migrationsbezogene Merkmale

Zur Beschreibung von Geschlechterunterschieden wurde das bei der Geburt in die Geburtsurkunde eingetragene Geschlecht (Selbstangabe) herangezogen. Auf Basis der schulischen und beruflichen Abschlüsse der Studienteilnehmenden wurde das Bildungsniveau anhand der Version 2011 der Internationalen Standardklassifikation für das Bildungswesen (*International Standard Classification of Education*, ISCED 2011 [25]) in niedrige (ISCED 0–2), mittlere (ISCED 3–4) und hohe (ISCED 5–8) Bildungsgruppen eingeteilt. Das Nettoäquivalenzeinkommen wurde anhand der Angaben der Teilnehmenden zur Höhe des monatlichen Nettoeinkommens ihrer Haushalte sowie der Haushaltszusammensetzung berechnet [26]. Fehlende Einkommensangaben wurden mittels regressionsanalytischer Verfahren mit Informationen zu Alter, Geschlecht, Erwerbsstatus, zur Haushaltszusammensetzung, Bildung, beruflichen Position sowie regionalen Informationen zur Arbeitslosigkeit und Einkommenssteuer imputiert. Nachfolgend wurden für die Auswertungen niedrige (Quintil 1), mittlere (Quintil 2–4) und hohe Einkommensgruppen (Quintil 5) gebildet.

Als migrationsbezogenes Merkmal wurde die Aufenthaltsdauer in Deutschland (anhand des Zuzugsjahres der Befragten) in die Angaben „bis 10 Jahre“, „11 bis 30 Jahre“, „31 Jahre und mehr“ sowie – bei in Deutschland geborenen Teilnehmenden – „seit Geburt“ kategorisiert. Der derzeitige Aufenthaltsstatus wurde anhand der folgenden Ausprägungen operationalisiert: „deutsche Staatsangehörigkeit“, „EU-Bürgerinnen und EU-Bürger“ und für Befragte mit syrischer oder türkischer Staatsangehörigkeit (als Drittstaatsangehörige) zudem „unbefristeter Aufenthaltsstatus“ und „befristeter Aufenthaltsstatus“.

Das Zugehörigkeitsgefühl zur Gesellschaft in Deutschland wurde mit der Frage erhoben: „Wie sehr fühlen Sie sich der Gesellschaft in Deutschland zugehörig?“ Die Antwortmöglichkeiten wurden für die Auswertungen in die folgenden drei Kategorien zusammengefasst: „sehr stark, stark“, „teils/teils“ und „kaum, gar nicht“. Darüber hinaus wurden selbstberichtete Diskriminierungserfahrungen in den Analysen berücksichtigt, welche wie folgt erhoben wurden: „Wie oft passiert Ihnen in Ihrem Alltag eines der folgenden Dinge?“ „Sie werden mit weniger Höflichkeit oder Respekt behandelt als andere“; „Sie erhalten einen schlechteren Service als andere Menschen (z. B. in Restaurants, Geschäften)“; „Jemand verhält sich so, als würde er oder sie Sie nicht ernst nehmen“; „Jemand verhält sich so, als hätte er oder sie Angst vor Ihnen“; „Sie werden bedroht oder belästigt.“ Teilnehmende, die in einem dieser Bereiche mit „sehr oft“, „oft“ oder „manchmal“ antworteten, wurden zusammengefasst und jenen gegenübergestellt, die über alle Kategorien hinweg „selten“ oder „nie“ angaben.

### Statistische Analysen

Es werden die Prävalenzen der jeweiligen nichtübertragbaren Erkrankungen nach verschiedenen sozialen und migrationsbezogenen Merkmalen mit entsprechenden 95 %-Konfidenzintervallen (95 %-KI) berichtet. Von einem statistisch signifikanten Unterschied zwischen Gruppen wird ausgegangen, wenn der aus dem jeweiligen Chi-Quadrat-Test ermittelte *p*-Wert kleiner als 0,05 ist.

Ergänzend zu den bivariaten Auswertungen wurden Poisson-Regressionsmodelle berechnet, um Zusammenhänge zwischen den einzelnen Erkrankungen und den sozialen sowie migrationsbezogenen Merkmalen zu untersuchen. Das Alter wurde in den Regressionsanalysen als metrische Variable (in Jahren) berücksichtigt. Ferner wurde in den Regressionsanalysen für die Staatsangehörigkeit nach Einwohnermeldeamt^3^ statistisch kontrolliert (adjustiert). Die mittels Poisson-Regressionen ermittelten *Prevalence Ratios* (PR) sowie die dazugehörigen 95 %-KI werden in den Ergebnissen als Forest-Plots dargestellt. Von statistisch signifikanten Assoziationen ist auszugehen, wenn die jeweiligen 95 %-KI den Wert 1 nicht einschließen. PR über 1 weisen auf eine höhere (unter 1 niedrigere) Prävalenz einer Erkrankung in der Gruppe hin, verglichen mit der Referenzgruppe.

In die Auswertungen wurde ein Gewichtungsfaktor einbezogen, der zum einen die Auswahlwahrscheinlichkeit der Studienpersonen in den Städten und Gemeinden, einschließlich der Auswahlwahrscheinlichkeit der Städte und Gemeinden an sich, berücksichtigt (Designgewichtung) und zum anderen die Stichprobe hinsichtlich folgender Merkmale an die Bevölkerung mit entsprechenden Staatsangehörigkeiten angleicht (Anpassungsgewichtung): Region, Geschlecht, Alter, Bildung und Aufenthaltsdauer. Diese Randverteilungen wurden dem Mikrozensus 2018 [27] entnommen, nachdem die Daten auf die ausgewählten fünf Staatsangehörigkeitsgruppen (einschließlich doppelter Staatsangehörigkeit) eingegrenzt wurden [20]. Fehlende Werte (Missings) in einzelnen untersuchten Variablen wurden in den Auswertungen ausgeschlossen.^4^

Alle Analysen wurden mit Stata/SE 17.0 (Stata Corp., College Station, TX, USA, 2017) unter Verwendung der Survey-Prozeduren für komplexe Stichproben durchgeführt [28].

## Ergebnisse

### Stichprobenbeschreibung

Die Studienpopulation ist in Tab. 1 dargestellt. Mehr als die Hälfte (53,8 %) der 6038 Teilnehmenden war männlich, der Altersmedian lag bei 41 Jahren (Range: 18–79 Jahre). 45,6 % der Befragten konnten der unteren und 14,3 % der oberen Bildungsgruppe zugeordnet werden. Der Median des monatlichen Nettoäquivalenzeinkommens lag bei 1190 €. Fast ein Drittel hatte eine Aufenthaltsdauer in Deutschland von 31 und mehr Jahren (29,1 %). In Bezug auf den Aufenthaltsstatus waren 40,9 % EU-Bürgerinnen und EU-Bürger. Fast zwei Drittel (63,8 %) der Befragten gaben an, sich der Gesellschaft in Deutschland (sehr) stark zugehörig zu fühlen. Von Diskriminierungserfahrungen im Alltag berichteten 41,2 % der Teilnehmenden.

**Table 1:** 

Merkmal	Fallzahl (*n*)	Relativer Anteil (in %, gewichtet)
**Geschlecht**		
Frauen	2983	46,2
Männer	3055	53,8
**Altersgruppen**		
18–39 Jahre	3024	46,5
40–59 Jahre	2156	37,9
60–79 Jahre	858	15,6
**Staatsangehörigkeit nach EMA**		
Italienisch	1205	18,9
Kroatisch	1223	18,0
Polnisch	1193	21,5
Syrisch	1209	15,4
Türkisch	1208	26,2
**Bildungsniveau (ISCED 2011)**		
Niedrig	1704	45,6
Mittel	2260	40,1
Hoch	2042	14,3
*Fehlende Angabe*	32	–
**Einkommen**		
Niedrig	1045	20,0
Mittel	3559	51,0
Hoch	1364	19,0
*Fehlende Angabe*	70	–
**Aufenthaltsdauer**		
Bis 10 Jahre	2474	28,0
11 bis 30 Jahre	1003	23,1
31 Jahre und mehr	1285	29,1
Seit Geburt	1189	19,8
*Fehlende Angabe*	87	–
**Aufenthaltsstatus**		
Deutsche Staatsangehörigkeit	1563	27,8
EU-Bürger:in	2568	40,9
Unbefristeter Aufenthalt	818	17,7
Befristeter Aufenthalt	1025	13,6
*Fehlende Angabe*	64	–
**Zugehörigkeitsgefühl Gesellschaft in DE**		
Sehr stark/stark	3655	63,8
Teils/teils	1824	29,1
Kaum/gar nicht	493	7,1
*Fehlende Angabe*	66	–
**Diskriminierungserfahrungen im Alltag**		
Ja	2464	41,2
Nein	3560	58,8
*Fehlende Angabe*	14	–

*n* ungewichtet, *ISCED* International Standard Classification of Education 2011 [25], *EMA* Einwohnermeldeamt, *DE* Deutschland

### Gesundheitliche Lage und damit assoziierte Faktoren

**Figure 1:**
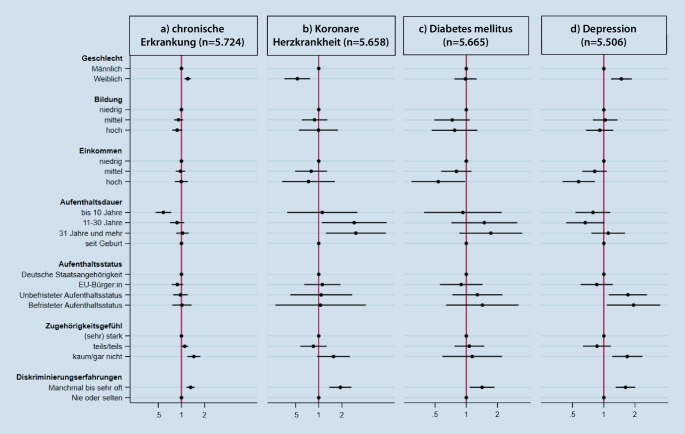

Die Prävalenzen der einzelnen nichtübertragbaren Erkrankungen variieren – teils in starkem Maße – nach den ausgewählten sozialen und migrationsbezogenen Merkmalen (Tab. 2). Nachfolgend liegt der Schwerpunkt auf den Ergebnissen der multivariablen Poisson-Regressionsanalysen (Abb. 1; Tab. 3,^5^).

**Table 2:** 

	Chronische Erkrankung bzw. lang andauerndes gesundheitliches Problem		Koronare Herzkrankheit		Diabetes mellitus		Depression	
	*n*	% (95 %-KI)	*n*	% (95 %-KI)	*n*	% (95 %-KI)	*n*	% (95 %-KI)
*Insgesamt*	2121/6019	37,7 (35,4–40,1)	267/5947	5,4 (4,3–6,6)	441/5955	8,4 (7,2–9,8)	793/5782	14,3 (12,8–16,0)
*Geschlecht*								
Frauen Männer *p*-Wert	1138/2970 983/3049	41,8 (38,7–45,0) 34,2 (31,5–37,0) **<** **0,001**	94/2941 173/3006	4,1 (3,0–5,6) 6,4 (5,1–8,2) **0,011**	213/2945 228/3010	8,2 (6,6–10,1) 8,6 (7,0–10,4) 0,745	458/2875 335/2907	17,0 (14,4–20,0) 12,0 (10,3–13,9) **0,004**
*Altersgruppen*								
18–39 Jahre 40–59 Jahre Ab 60 Jahre *p*-Wert	690/3022 897/2149 534/848	25,0 (22,5–27,8) 42,8 (39,5–46,3) 63,4 (57,6–68,8) **<** **0,001**	24/2988 92/2122 151/837	1,0 (0,5–2,0) 4,9 (3,6–6,6) 19,8 (15,7–24,6) **<** **0,001**	73/2978 174/2125 194/852	3,0 (2,1–4,2) 8,6 (6,7–10,9) 23,9 (19,8–28,5) **<** **0,001**	296/2872 326/2088 171/822	11,0 (9,5–12,8) 15,1 (12,4–18,1) 22,4 (18,0–27,5) **<** **0,001**
*Bildung (ISCED 2011)*								
Niedrig Mittel Hoch *p*-Wert	677/1700 780/2252 651/2036	42,2 (38,3–46,2) 34,7 (31,4–38,2) 32,0 (28,5–35,8) **<** **0,001**	112/1668 91/2223 57/2024	6,9 (5,3–9,0) 4,1 (3,0–5,6) 3,6 (2,0–6,4) **0,008**	212/1679 128/2224 95/2020	12,3 (10,0–15,0) 5,2 (3,6–6,8) 5,0 (3,3–7,6) **<** **0,001**	242/1630 287/2162 261/1959	15,6 (13,0–18,7) 13,7 (11,6–16,1) 12,0 (9,3–15,2) 0,218
*Einkommen*								
Niedrig Mittel Hoch *p*-Wert	377/1040 1.298/3548 414/1362	38,7 (34,1–43,5) 38,6 (35,5–41,7) 33,7 (29,2–38,4) 0,189	55/1018 175/3506 31/1354	5,9 (3,9–9,0) 5,9 (4,7–7,4) 3,0 (1,6–5,4) 0,069	101/1025 285/3507 45/1354	10,5 (7,7–14,1) 9,0 (7,4–10,8) 3,9 (2,6–6,0) **<** **0,001**	157/981 482/3405 144/1327	17,6 (14,5–21,1) 14,9 (12,9–17,1) 9,1 (6,7–12,2) **<** **0,001**
*Aufenthaltsdauer*								
Bis 10 Jahre 11 bis 30 Jahre 31 Jahre und mehr Seit Geburt *p*-Wert	623/2471 377/1000 731/1275 358/1187	22,5 (20,0–25,3) 35,6 (30,7–40,8) 57,5 (53,2–61,6) 32,7 (28,5–37,1) **<** **0,001**	57/2440 41/991 150/1254 8/1176	1,1 (0,7–1,9) 4,8 (3,1–7,3) 12,9 (10,3–15,9) 0,6 (0,2–1,4) **<** **0,001**	117/2434 71/991 201/1269 30/1177	3,7 (2,7–5,2) 7,3 (5,3–10,0) 17,1 (14,2–20,4) 2,8 (1,6–4,9) **<** **0,001**	233/2332 135/968 260/1249 153/1149	11,4 (9,2–14,0) 10,8 (8,3–13,9) 21,2 (17,7–25,1) 12,5 (9,8–15,8) **<** **0,001**
*Aufenthaltsstatus*								
Deutsche Staatsangehörigkeit EU-Bürger:in Unbefristeter Aufenthalt Befristeter Aufenthalt *p*-Wert	601/1554 893/2561 318/815 290/1025	41,1 (37,1–45,3) 38,3 (34,9–41,8) 40,5 (35,0–46,3) 26,2 (21,6–31,4) **<** **0,001**	59/1540 109/2533 63/808 33/1005	4,6 (3,1–6,8) 5,7 (4,3–7,7) 8,8 (6,2–12,5) 1,7 (0,8–3,3) **<** **0,001**	86/1544 171/2538 104/806 73/1005	6,4 (4,8–8,4) 8,5 (6,8–10,6) 13,4 (9,9–17,8) 5,8 (3,7–9,0) **<** **0,001**	210/1516 346/2484 135/782 96/944	14,2 (11,5–17,5) 13,3 (11,3–15,7) 17,8 (13,7–22,7) 13,2 (9,4–18,3) 0,258
*Zugehörigkeitsgefühl Gesellschaft in DE*								
Sehr stark/stark Teils/teils Kaum/gar nicht *p*-Wert	1258/3644 640/1817 197/492	35,9 (32,9–38,9) 39,0 (35,5–42,7) 46,2 (37,8–54,8) **0,035**	169/3604 72/1798 21/482	5,0 (3,9–6,4) 5,0 (3,5–7,1) 8,5 (4,8–14,6) 0,176	279/3613 132/1795 26/482	8,0 (6,4–9,8) 9,2 (7,2–11,7) 9,5 (5,3–16,4) 0,612	464/3543 225/1713 95/468	13,5 (11,5–15,7) 13,5 (10,7–17,0) 24,7 (18,6–32,1) **0,004**
*Diskriminierungserfahrungen im Alltag*								
Ja Nein *p*-Wert	963/2458 1149/3547	43,2 (39,7–46,7) 33,8 (30,7–37,1) **<** **0,001**	121/2411 141/3523	6,7 (5,2–8,8) 4,4 (3,3–5,7) **0,007**	181/2414 258/3527	9,4 (7,5–11,8) 7,7 (6,3–9,4) 0,1770	409/2317 380/3452	18,6 (16,1–21,3) 11,4 (9,8–13,3) **<** **0,001**

*n* ungewichtet, *%* gewichtet, *95* *%-KI* 95 %-Konfidenzintervall, *DE* Deutschland, *EU* Europäische Union, *ISCED 2011* International Standard Classification of Education 2011 [25]

*Fettgedruckte p‑Werte* statistisch signifikant gemäß Chi-Quadrat-Test

**Table 3:** 

	Chronische Erkrankung bzw. lang andauerndes gesundheitliches Problem (*n* = 5724)		Koronare Herzkrankheit (*n* = 5658)		Diabetes mellitus (*n* = 5665)		Depression (*n* = 5506)	
	PR (95 %-KI)	*p*-Wert	PR (95 %-KI)	*p*-Wert	PR (95 %-KI)	*p*-Wert	PR (95 %-KI)	*p*-Wert
*Geschlecht*								
Frauen	1,21 (1,09–1,33)	**<** **0,001**	0,53 (0,36–0,77)	**0,001**	0,98 (0,77–1,26)	0,893	1,48 (1,17–1,86)	**0,001**
Männer (Ref.)								
*Bildung (ISCED 2011)*								
Niedrig (Ref.)								
Mittel	0,92 (0,80–1,05)	0,221	0,88 (0,60–1,29)	0,522	0,73 (0,49–1,08)	0,126	1,03 (0,78–1,36)	0,823
Hoch	0,88 (0,76–1,02)	0,092	0,99 (0,55–1,78)	0,975	0,77 (0,46–1,28)	0,347	0,91 (0,68–1,23)	0,550
*Einkommen*								
Niedrig (Ref.)								
Mittel	0,97 (0,84–1,12)	0,697	0,80 (0,49–1,29)	0,350	0,80 (0,57–1,12)	0,196	0,81 (0,62–1,07)	0,137
Hoch	1,0 (0,82–1,21)	0,976	0,74 (0,33–1,62)	0,442	0,54 (0,30–0,97)	**0,041**	0,57 (0,40–0,82)	**0,003**
*Aufenthaltsdauer*								
Bis 10 Jahre	0,58 (0,46–0,74)	**<** **0,001**	1,11 (0,39–3,16)	0,845	0,93 (0,39–2,19)	0,860	0,79 (0,53–1,15)	0,215
11 bis 30 Jahre	0,88 (0,71–1,09)	0,225	2,88 (1,09–7,60)	**0,032**	1,49 (0,72–3,10)	0,283	0,66 (0,44–1,01)	0,058
31 Jahre und mehr	1,03 (0,86–1,25)	0,725	3,03 (1,24–7,41)	**0,016**	1,72 (0,85–3,45)	0,127	1,10 (0,76–1,60)	0,605
Seit Geburt (Ref.)								
*Aufenthaltsstatus*								
Deutsche Staatsangehörigkeit (Ref.)								
EU-Bürger:in	0,89 (0,75–1,05)	0,162	1,11 (0,65–1,91)	0,693	0,89 (0,56–1,43)	0,637	0,85 (0,60–1,22)	0,383
Unbefristeter Aufenthalt	0,97 (0,78–1,21)	0,805	1,08 (0,43–2,71)	0,875	1,28 (0,72–3,10)	0,381	1,71 (1,12–2,61)	**0,014**
Befristeter Aufenthalt	1,02 (0,76–1,36)	0,912	1,05 (0,27–4,07)	0,938	1,43 (0,64–3,19)	0,380	1,93 (1,07–3,50)	**0,030**
*Zugehörigkeitsgefühl Gesellschaft in DE*								
Sehr stark/stark (Ref.)								
Teils/teils	1,10 (0,99–1,22)	0,073	0,85 (0,58–1,27)	0,429	1,07 (0,77–1,49)	0,687	0,86 (0,63–1,17)	0,328
Kaum/gar nicht	1,45 (1,20–1,76)	**<** **0,001**	1,56 (0,95–2,55)	0,078	1,14 (0,59–2,21)	0,695	1,68 (1,20–2,36)	**0,003**
*Diskriminierungserfahrungen im Alltag*								
Ja	1,31 (1,16–1,48)	**<** **0,001**	1,91 (1,37–2,66)	**<** **0,001**	1,42 (1,08–1,87)	**0,013**	1,62 (1,30–2,02)	**<** **0,001**
Nein (Ref.)								

Lesebeispiel PR: Die Prävalenz einer chronischen Erkrankung oder eines lang andauernden gesundheitlichen Problems liegt bei Frauen um das 1,21-fache höher als bei Männern

*DE* Deutschland, *EU* Europäische Union, *ISCED 2011* International Standard Classification of Education 2011 [25], *PR* Prevalence Ratio, *Ref.* Referenzgruppe

*Fettdruck* statistisch signifikant im Vergleich zur Referenzgruppe, adjustiert für Alter (metrisch) und Staatsangehörigkeit nach Einwohnermeldeamt

#### Chronische Krankheit oder lang andauerndes gesundheitliches Problem

Über ein Drittel (37,7 %) der Befragten gab an, eine chronische Krankheit oder ein lang andauerndes gesundheitliches Problem zu haben. Die Ergebnisse der Poisson-Regressionsanalyse zeigen (Abb. 1a), dass – nach wechselseitiger Adjustierung der sozialen sowie migrationsbezogenen Merkmale – ein weibliches Geschlecht mit einer höheren Prävalenz assoziiert ist. Im Hinblick auf die migrationsbezogenen Merkmale zeigt sich zudem ein Zusammenhang zwischen der Aufenthaltsdauer und dem Vorliegen einer chronischen Krankheit oder eines lang andauernden gesundheitlichen Problems: Befragte mit einer Aufenthaltsdauer von bis zu 10 Jahren berichteten seltener davon als Teilnehmende, die seit ihrer Geburt in Deutschland leben. Darüber hinaus sind ein als gering empfundenes Zugehörigkeitsgefühl zur Gesellschaft in Deutschland und selbstberichtete Diskriminierungserfahrungen im Alltag mit einer höheren Prävalenz einer chronischen Erkrankung oder eines lang andauernden gesundheitlichen Problems assoziiert.

#### Koronare Herzkrankheit

Insgesamt 5,4 % der Teilnehmenden gaben an, dass bei ihnen jemals eine koronare Herzkrankheit ärztlich diagnostiziert wurde. Unter statistischer Konstanthaltung der verschiedenen sozialen sowie migrationsbezogenen Merkmale (Abb. 1b) ist festzustellen, dass ein weibliches Geschlecht mit einer geringeren Prävalenz und – gegenüber in Deutschland geborenen Teilnehmenden – eine Aufenthaltsdauer von 11 bis 30 Jahren sowie 31 Jahren oder länger mit einer höheren Prävalenz einer koronaren Herzkrankheit assoziiert sind. So liegt das Risiko einer koronaren Herzerkrankung bei Teilnehmenden mit einer Aufenthaltsdauer von 31 und mehr Jahren knapp 3‑mal so hoch wie bei Befragten, die seit ihrer Geburt in Deutschland leben. Außerdem gaben Befragte, die von Diskriminierungserfahrungen berichteten, häufiger das Vorliegen einer jemals ärztlich diagnostizierten koronaren Herzerkrankung an.

#### Diabetes mellitus

Die Prävalenz eines selbstberichteten ärztlich diagnostizierten Diabetes mellitus lag bei insgesamt 8,4 %. Nach multivariabler Adjustierung in der Poisson-Regressionsanalyse zeigt sich (Abb. 1c), dass Befragte mit einem hohen Einkommen eine geringere Diabetesprävalenz aufweisen als jene mit niedrigerem Einkommen. Selbstberichtete Diskriminierungserfahrungen im Alltag sind zudem mit einer höheren Prävalenz eines ärztlich diagnostizierten Diabetes mellitus assoziiert.

#### Depression

Insgesamt 14,3 % der Teilnehmenden berichteten, dass bei ihnen jemals eine Depression ärztlich diagnostiziert wurde. Die Ergebnisse der multivariablen Poisson-Regressionsanalyse zeigen, dass ein weibliches Geschlecht häufiger mit einer selbstberichteten ärztlich diagnostizierten Depression einhergeht. Dagegen berichteten Teilnehmende mit einem hohen Einkommen seltener von dem Vorliegen einer Depressionsdiagnose als jene mit niedrigerem Einkommen. Neben den sozialen Merkmalen ist die Prävalenz einer selbstberichteten ärztlich diagnostizierten Depression mit dem Aufenthaltsstatus assoziiert. Insbesondere bei Personen mit befristetem Aufenthaltsstatus liegt das Risiko fast doppelt so hoch wie bei jenen mit deutscher Staatsangehörigkeit. Ferner sind ein geringes Zugehörigkeitsgefühl zur Gesellschaft in Deutschland und selbstberichtete Diskriminierungserfahrungen im Alltag mit höheren Prävalenzen einer selbstberichteten ärztlich diagnostizierten Depression assoziiert.

## Diskussion

Die vorliegenden Analysen nehmen wichtige nichtübertragbare Erkrankungen bei 18- bis 79-jährigen in Deutschland lebenden Menschen mit italienischer, kroatischer, polnischer, syrischer oder türkischer Staatsangehörigkeit in den Blick. Zum einen wurden Prävalenzen selbstberichteter Morbidität ermittelt, zum anderen Zusammenhänge zwischen den nichtübertragbaren Erkrankungen und verschiedenen sozialen sowie migrationsbezogenen Merkmalen differenziert untersucht. Dabei waren insbesondere selbstberichtete Diskriminierungserfahrungen im Alltag und zum Teil auch ein geringeres Zugehörigkeitsgefühl zur Gesellschaft in Deutschland mit erhöhten Prävalenzen der nichtübertragbaren Erkrankungen assoziiert.

### Prävalenzen von selbstberichteten nichtübertragbaren Erkrankungen bei Menschen mit ausgewählten Staatsangehörigkeiten

Ein Drittel aller Teilnehmenden gab an, eine chronische Krankheit oder ein lang andauerndes gesundheitliches Problem zu haben. Dieser Anteil liegt, zumindest für die jüngeren Altersgruppen deutlich unter dem, der für die erwachsene Bevölkerung in Deutschland (48 %) anhand der deutschsprachigen telefonischen Querschnittsbefragung GEDA 2019/2020-EHIS ermittelt wurde.^6^ In der Gruppe der über 65-Jährigen ist die Prävalenz einer selbstberichteten chronischen Krankheit in beiden Befragungsstudien hingegen nahezu identisch. Für die drei ausgewählten selbstberichteten ärztlichen Diagnosen koronare Herzkrankheit, Diabetes und Depression liegen Unterschiede bezüglich der Operationalisierung der Erkrankungen in den Studien vor.^7^ Aus diesem Grund wird auf einen Vergleich der Prävalenzen verzichtet. Im Rahmen von GEDA Fokus wurde bei rund 5 % der Teilnehmenden jemals eine koronare Herzkrankheit sowie bei 8 % der Teilnehmenden ein Diabetes mellitus ärztlich diagnostiziert. Die Lebenszeitprävalenz einer selbstberichteten ärztlich diagnostizierten Depression lag bei knapp 14 %.

### Assoziationen zwischen nichtübertragbaren Erkrankungen und sozialen sowie migrationsbezogenen Merkmalen

Die Studie GEDA Fokus bietet in besonderer Art und Weise die Möglichkeit, Zusammenhänge zwischen gesundheitsrelevanten Indikatoren und verschiedenen sozialen sowie migrationsbezogenen Merkmalen differenziert zu untersuchen. Im Hinblick auf die soziodemografischen Faktoren zeigen sich in multivariablen Analysen vergleichbare Geschlechtsunterschiede für die Studienteilnehmenden in GEDA Fokus, wie sie schon in GEDA 2019/2020-EHIS gezeigt wurden [29]. Zudem deuten die Poisson-Regressionsanalysen auf Einkommensunterschiede hin: Teilnehmende mit einem hohen Einkommen gaben – im Vergleich zu jenen mit niedrigerem Einkommen – seltener an, dass bei ihnen jemals ein Diabetes mellitus oder eine Depression ärztlich diagnostiziert wurde. Dies steht im Einklang mit dem – unabhängig vom Vorliegen einer Migrationsgesichte – empirisch bereits fundierten Zusammenhang zwischen sozialer Ungleichheit und dem Auftreten chronischer Erkrankungen [30].

Hinsichtlich der ausgewählten migrationsbezogenen Merkmale zeigen die Ergebnisse der Poisson-Regressionsanalysen, dass – bei Adjustierung für das Alter – eine kürzere Aufenthaltsdauer in Deutschland (von bis zu 10 Jahren) mit einer geringeren Prävalenz einer chronischen Erkrankung oder eines lang andauernden gesundheitlichen Problems assoziiert ist, eine Aufenthaltsdauer ab 11 Jahren mit einer höheren Prävalenz einer ärztlich diagnostizierten koronaren Herzerkrankung (im Vergleich zu in Deutschland geborenen Teilnehmenden). Dies steht im Einklang mit dem sogenannten Healthy Migrant Effect, wonach vor allem junge und gesunde Menschen migrieren, die gegenüber der Bevölkerung des Ziellandes einen gesundheitlichen Vorteil aufweisen. Gesundheitliche Risiken für chronische nichtübertragbare Erkrankungen konvergieren jedoch über die Zeit [2, 31]. Dies wird unter anderem auf eine geringere Inanspruchnahme von Leistungen des Gesundheitssystems (infolge sozialer sowie struktureller Barrieren) und eine mögliche sozioökonomische Benachteiligung zurückgeführt. Zudem kann die Ausübung gesundheitsgefährdender beruflicher Tätigkeiten, wie solche mit einer hohen körperlichen Arbeitsbelastung über einen langen Zeitraum hinweg, eine höhere Vulnerabilität in Bezug auf chronische Erkrankungen schaffen [2, 31, 32]. Menschen mit eigener Migrationserfahrung sind in Deutschland beispielsweise häufiger in gesundheitsgefährdenden Berufen beschäftigt als Personen, die in Deutschland geboren wurden ([32]; siehe dazu auch den Beitrag von Kajikhina et al. in dieser Themenausgabe).

Ferner zeigte sich, dass der Aufenthaltsstatus mit einer selbstberichteten jemals ärztlich diagnostizierten Depression assoziiert ist. So weisen Befragte mit einem unbefristeten Aufenthaltsstatus ein um 70 % höheres Risiko und Befragte mit einem befristeten Aufenthaltsstatus sogar ein fast doppelt so hohes Risiko einer Depression auf als jene mit deutscher Staatsangehörigkeit. Studienteilnehmende mit einem befristeten Aufenthaltsstatus gaben in GEDA Fokus am häufigsten als Hauptgründe für den Zuzug nach Deutschland Krieg und Verfolgung an.^8^ Menschen mit Fluchterfahrung sind einem höheren Risiko für eine schlechtere psychische Gesundheit ausgesetzt: Neben traumatisierenden Erlebnissen vor, während und nach der Flucht sind – Studien zufolge – „postmigratorische Faktoren“, wie Erwerbslosigkeit, Einsamkeit und ein abgelehnter oder noch nicht entschiedener Asylantrag mit dem Vorliegen von depressiven Symptomen bei Menschen mit Fluchterfahrung assoziiert [6].

Im Einklang mit bisherigen Studien zeigt sich weiterhin, dass ein als gering empfundenes Zugehörigkeitsgefühl zur Gesellschaft in Deutschland und selbstberichtete Diskriminierungserfahrungen mit einer schlechteren physischen sowie psychischen Gesundheit assoziiert sind [33–37]. Internationale Studien verweisen zudem auf einen Zusammenhang zwischen Diskriminierungserfahrungen im Alltag und Herz-Kreislauf-Erkrankungen sowie Diabetes mellitus [38–40]. Ausgrenzungsprozesse können nicht nur als psychosoziale Stressoren direkt auf die Gesundheit wirken, sondern auch indirekt, indem sie einen geringeren Ressourcenzugang indizieren: Neben interpersoneller Diskriminierung können strukturelle sowie institutionelle Diskriminierungsrisiken eine gleichberechtigte Teilhabe im Gesundheitssystem erschweren [2, 41, 42]. So sind beispielsweise Asylsuchende infolge des aufenthaltsrechtlichen Status mit gesetzlichen Einschränkungen (§§ 4, 6 Asylbewerberleistungsgesetz) im Anspruch auf die gesundheitliche Versorgung und mit einem eingeschränkten Umfang der Leistungen konfrontiert [42, 43]. Darüber hinaus stellen z. B. auch die sprachlich homogenen Versorgungsstrukturen des Gesundheitswesens institutionelle Diskriminierungsrisiken dar, die den Zugang zur und die Qualität der gesundheitlichen Versorgung beeinflussen können ([41]; siehe auch den Beitrag von Kajikhina et al. in dieser Ausgabe).

Zum Ausmaß, den Formen und Auswirkungen individueller sowie institutioneller Diskriminierung bedarf es sowohl bezüglich des Zugangs zum Gesundheitssystem als auch der Qualität der gesundheitlichen Versorgung weiterer differenzierter Analysen. Zudem sind Erkenntnisse darüber erforderlich, inwiefern Diskriminierungs- und Ausgrenzungserfahrungen im Gesundheitssystem verschiedene Gesundheitsoutcomes beeinflussen und somit gesundheitliche Ungleichheiten verstärken.

## Stärken und Limitationen

Die Befragungsstudie GEDA Fokus ermöglicht differenzierte Analysen zu unterschiedlichen gesundheitsrelevanten Faktoren anhand einer großen Stichprobe von Menschen mit italienischer, kroatischer, polnischer, syrischer oder türkischer Staatsangehörigkeit aus ganz Deutschland.

Limitierend ist jedoch anzumerken, dass die Auswahl der Stichprobe einzig dem Merkmal der Staatsangehörigkeit unterlag und damit große Subgruppen unter der heterogenen Gruppe der Menschen mit Migrationsgeschichte von der Befragung ausgeschlossen waren (z. B. eingebürgerte Personen mit ausschließlich deutscher Staatsangehörigkeit bzw. Personen, deren Staatsangehörigkeit eine andere als die der fünf ausgewählten ist). Rückschlüsse der Ergebnisse auf die Gruppe *aller* Menschen mit Migrationsgeschichte in Deutschland sind demnach nicht möglich. Darüber hinaus sind die Fallzahlen für differenzierte Betrachtungen einzelner Indikatoren zur Beschreibung der gesundheitlichen Lage sehr niedrig, wodurch die Interpretation teilweise eingeschränkt ist. Weiterhin gilt es zu berücksichtigen, dass anhand der vorliegenden Querschnittsdaten keinerlei Rückschlüsse hinsichtlich der Wirkrichtung von Zusammenhängen möglich sind. Entsprechend wichtig ist es, die beobachteten Zusammenhänge auch langfristig in Kohortenstudien zu untersuchen. Zudem unterliegen selbstberichtete Diagnosen als Indikatoren unter anderem einem Inanspruchnahme-, einem Recall- und einem Reporting-Bias [44]. Ihre Übereinstimmung mit tatsächlich vergebenen NCD-Diagnosen ist unterschiedlich gut (z. B. [45, 46]), zumal sie gerade in Bezug auf psychische Krankheiten (wie Depressionen) Morbidität nur beschränkt darstellen [47].

## Fazit

Unsere Ergebnisse zeigen, dass insbesondere selbstberichtete Diskriminierungserfahrungen im Alltag und zum Teil auch ein geringeres Zugehörigkeitsgefühl zur Gesellschaft in Deutschland mit erhöhten Prävalenzen verschiedener nichtübertragbarer Erkrankungen assoziiert sind. Um gesundheitlicher Ungleichheit nachhaltig begegnen zu können, bedarf es der systematischen Erforschung der Formen und Auswirkungen gesellschaftlicher und systemischer Teilhabebarrieren. Darüber hinaus bedarf es der Berücksichtigung des gesamten Lebenslaufs bei der Analyse von Mustern chronischer Erkrankungen: So wirken vor, während und insbesondere nach der Migration unterschiedliche lebensweltbezogene Expositionen und Ausschlussmechanismen auf die Gesundheit, die sowohl direkt als auch nach langer Latenzzeit das Risiko für bestimmte chronische Erkrankungen erhöhen können [2, 3].

## Footnotes

1 Hausbesuche wurden aus logistischen Gründen nur in den 53 großen Städten, nicht jedoch in den 46 kleineren Gemeinden angeboten.

2 Diese wurde in der Frageformulierung erklärt als „eine Durchblutungsstörung am Herzen, eine Verengung der Herzkranzgefäße oder eine Angina pectoris“.

3 Auf die Ausweisung von Ergebnissen nach Staatsangehörigkeitsgruppen wird jedoch verzichtet, da sich zum einen die Stichprobenzusammensetzung systematisch zwischen den einzelnen Gruppen unterscheidet und eine Vergleichbarkeit dieser daher erschwert ist (z. B. haben Menschen mit italienischer Staatsangehörigkeit häufiger selbstinitiativ zu Studienbeginn teilgenommen, durch Hausbesuche im späteren Studienverlauf wurden jedoch häufiger Menschen mit niedrigerer Bildung und einer schlechteren subjektiven Gesundheit erreicht). Zum anderen birgt der Vergleich die Gefahr, bei Beschreibung einzelner Effekte je nach Staatsangehörigkeit pauschalisierend und stereotypisierend zu sein.

4 Separate Missing-Kategorien wurden aufgrund eines möglicherweise systematisch verzerrten Antwortverhaltens nicht mitgeführt, zudem sind diese Kategorien unzureichend interpretierbar.

5 Der Tabelle können die Prevalence Ratios und entsprechenden 95 %-Konfidenzintervalle sowie *p*-Werte der ausgewählten Erkrankungen nach den sozialen und migrationsbezogenen Merkmalen entnommen werden.

6 Eigene Berechnungen, da die Grundgesamtheit der GEDA-2019/2020-EHIS-Befragung auch Personen ab 80 Jahren einschließt.

7 Für einen Überblick der Operationalisierung der Erkrankungen in GEDA 2019/2020-EHIS siehe Heidemann et al. [29] .

8 Eigene Berechnungen.
